# Insight into the interactions of fullerenes with biological membranes through molecular dynamics simulations

**DOI:** 10.1080/23746149.2024.2350160

**Published:** 2024-05-10

**Authors:** Nililla Nisoh, Viwan Jarerattanachat, Cristiano Dias, Jirasak Wong-Ekkabut

**Affiliations:** a Department of Physics, Faculty of Science, Kasetsart University, Bangkok, Thailand;; b Computational Biomodelling Laboratory for Agricultural Science and Technology (CBLAST), Faculty of Science, Kasetsart University, Bangkok, Thailand;; c Thailand Center of Excellence in Physics (ThEP Center), Ministry of Higher Education, Science, Research and Innovation, Bangkok, Thailand;; d NSTDA Supercomputer Center (ThaiSC), National Electronics and Computer Technology Center (NECTEC), National Science and Technology Development Agency (NSTDA), Pathumthani, Thailand;; e Department of Physics, New Jersey Institute of Technology, Newark, NJ, USA

**Keywords:** Fullerene, lipid bilayer, Molecular dynamics simulations, biological membrane

## Abstract

This paper reviews our current understanding of how fullerene molecules interact with biological membranes. First, we discuss how fullerenes permeate into biological membranes. Next, the putative mechanisms of fullerene aggregation and dispersion within lipid bilayers are discussed along with their potential toxic effect on biological membranes. Finally, current trends in the study of fullerene-membrane interactions are highlighted as well as future challenges that need to be overcome to further advance the field.

## GRAPHICAL ABSTRACT

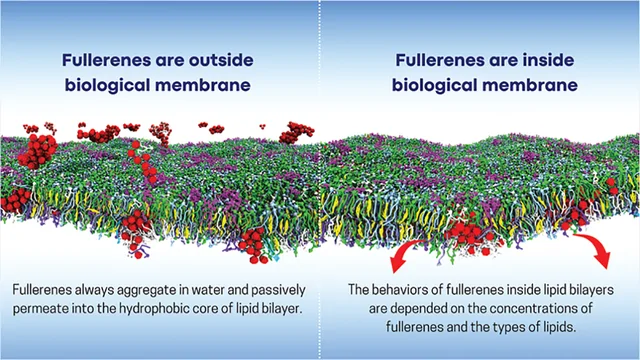

## Introduction

Since its discovery in 1985, buckminsterfullerene (C_60_) has been the most renowned molecule among the extensive family of fullerenes [1]. It has illicit great interest in a wide range of fields, including nanoelectronics, energy, optics, pharmaceutical and biomedical applications [2–5]. This is due to fullerene’s unique physical and chemical properties which include high membrane permeability, antioxidant activity, and biocompatibility. These properties are suitable for various applications including drug delivery systems, cancer therapy and diagnosis, and bioimaging [6–9]. However, fullerenes are also emitted into the environment as a result of industrial and natural processes produced by fuel combustion and heating plants [10–13]. This increased presence of fullerenes is raising concerns about their potential toxicity to humans.

Fullerenes have been found to permeate cell membranes, which allows them to cross olfactory nerves, the blood–brain barrier, and lung surfactant [14–18]. However, some of these fullerenes are retained within the cell membrane disrupting its integrity, which may account for the toxicity and safety levels of fullerenes to humans. Accordingly, extensive studies are being performed to shed light into the bio-hazardous and toxic effects of fullerenes in lipid membranes. Molecular dynamics (MD) simulations can provide detailed insights into the permeation and aggregation of fullerenes, as well as the shape of fullerene clusters inside bilayers. By varying the concentration of fullerenes and the lipid composition of membranes, researchers can investigate how these factors affect the behavior of fullerenes within the bilayer and how the presence of fullerenes affects the structural and dynamical properties of the lipid bilayers. Overall, this is an active area of research, and a better understanding of the fullerene-membrane interactions could potentially lead to novel applications in biomedicine and help to determine the toxicity of fullerenes in living cells. However, the complexity and diversity of real biological systems make it challenging to accurately model the interactions of fullerenes with complex membranes. Recently, a model of the plasma membrane has been used for that purpose [19].

This article reviews experimental and computational studies of fullerene permeation into biological membranes. The primary emphasis of this review is on C_60_, especially in the context of computational simulations. However, other fullerenes, such as C_180_, C_540_, fullerenes with polar functional groups, and Janus fullerene, are also partially addressed within this article. We explain how C_60_ interacts with lipid membranes, including their behavior after permeating inside the membrane. Additionally, we address the mechanisms of C_60_ aggregation and dispersion within lipid bilayers and discuss the potential toxic effects of fullerenes on biological membranes. Finally, we present perspective trends and future directions for studying the interaction of fullerenes with biological membranes.

## Fullerene penetration in the biological membranes

The release of fullerenes into the environment has been identified as a potential risk to human health [15,21–23]. Because of its hydrophobicity, fullerenes can enter and potentially disrupt biological membranes. It is therefore important to understand how fullerenes penetrate and localize within biological membranes, and how they interact within the cell. These fundamental studies can determine fullerene’s toxicity and its potential application for drug delivery.

Many experimental techniques have been employed to study how fullerene penetrates living cells. Porter et al. used electron microscopy to show that fullerenes permeate the cell membrane to become localized within the cytoplasm, lysosomes, and cell nuclei of human monocyte-derived macrophage cells [17]. Later, Russ et al. also suggested that fullerenes entered directly into the cells of mouse macrophages RAW 264.7 through endocytosis/pinocytosis, and passive diffusion [24]. Moreover, confocal microscopy and fluorescence analysis revealed the permeation of fullerenes into leukemic cells [25], the endoplasmic reticulum of human mast cells [14], and the plasma membrane [26]. These experimental results demonstrate that fullerenes directly penetrate lipid membranes. However, little is known about the entry mechanism, as well as the structure and dynamical organization of fullerene in the cellular membrane. Therefore, computer simulations have been intensively utilized to provide this type of information at the molecular level.

Molecular dynamics (MD) simulations can shed light into the atomic interactions taking place between different components of a biological system. The cell membrane is one of the most fundamental structural elements of biological systems, which is often modeled as a lipid bilayer. Although realistic biological membranes are complex multicomponent systems, several molecular dynamics (MD) studies have focused on fullerenes in both the single-lipid type of bilayer [27–35] and the binary mixture bilayers [36,37]. To mimic most realistic cellular membranes, computer simulations need assistance from experiments to develop novel membrane models. Over the last decade, all-atom MD simulations have been successfully used to study the passive transport of a fullerene from water into single-lipid membranes such as DPPC [27] and DMPC lipid bilayers [28,38]. It was shown that fullerene easily penetrates into the lipid bilayer within a few milliseconds [27]. In a study done by Li et al. the preferred location of a fullerene in the bilayer was investigated, discovering a fullerene preference at about 0.6–0.7 nm away from DMPC bilayer center [28].

**Figure 1.**
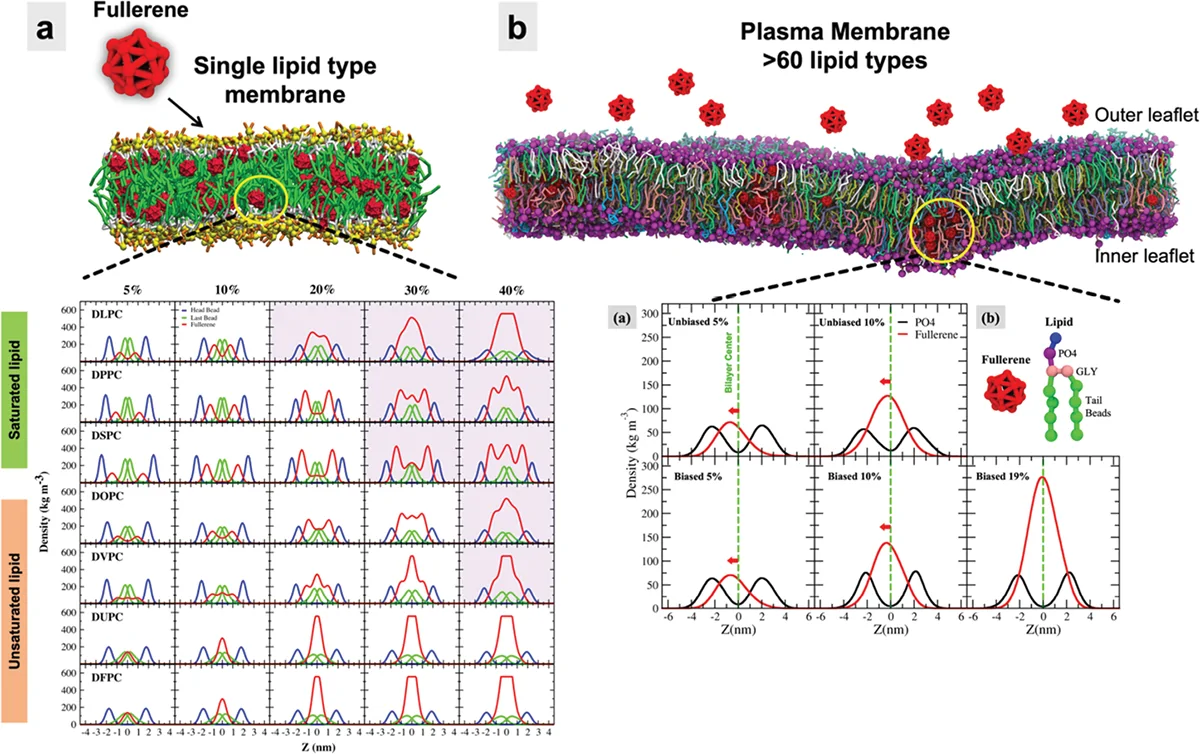
Penetration of fullerenes into lipid membranes. Panel (a) illustrates a snapshot of fullerenes in a single lipid type membrane and a graph of the density profile of different lipid components (blue and green) and fullerenes (red) as a function of the bilayer normal (z-axis) at varying fullerene concentrations in saturated and unsaturated bilayers. Panel (b) shows a snapshot of fullerene in the plasma membrane and a graph of density profiles of phosphate groups (black) and fullerenes (red) for plasma membrane simulations, indicating that fullerenes are preferentially located in the inner leaflet region. (a) and (b) adapted from Nisoh et al [19,20].

Fullerene is known to aggregate and form stable clusters in an aqueous solution. The ability of fullerene clusters to penetrate into different membranes was investigated using coarse-grained (CG) MD simulations [30,34,36,39,40]. The Martini CG model, which groups three or four non-hydrogen atoms as a single particle, allows for the simulation of larger systems over longer timescales compared to all-atom simulations [41,42]. Several studies have reported that fullerene clusters can passively translocate into various membrane models (Figure 1(a)) including single lipid types (DPPC, DOPC) [30,34,35,39] and lipid membrane mixtures such as the one modeling the epidermis [40], bacteria membranes [33], peroxidized lipid membranes [37], and plasma membranes [19]. Gul et al. recently demonstrated that fullerene permeates more rapidly into fully peroxidized lipid bilayer than pure lipid bilayer membranes [37]. In a recent study, Ingolfsson et al. developed a CG model of the plasma membrane, which is asymmetric and accounts for more than 60 types of lipids [43]. This model enabled us to study fullerene interactions with plasma membranes [19]. Our recent study reveals that fullerenes rapidly aggregate into small clusters in the water phase and subsequently translocate the plasma membrane. Interestingly, fullerene clusters can penetrate deeply inside into the inner leaflet of the plasma membrane (Figure 1(b)).

Another interesting study is the transfer of different-sized fullerenes (C_60_, C_180_ and C_540_) into lipid membranes [29]. The study indicates that fullerenes with range size varying from 1.1 to 2.4 nm can translocate from the water phase to the center of the lipid bilayer. Tian et al. investigated the penetration of fullerene polymer chains containing both hydrophobic and hydrophilic segments into a lipid bilayer [44]. Fullerene polymer chains with hydrophobic segments required thermal activation energy to promote their penetration into the bilayer. In addition, fullerene polymer chains have been used in photodynamic cancer therapy by inducing cleavage of DNA in cancer cells causing cell death [45].

Despite the abundance of studies showing that fullerene successfully penetrate into the bilayer, some studies have reported the failure of penetration. Spurlin et al. showed that fullerenes adhered to lipid bilayers but do not penetrate into the lipid hydrocarbon chains. No effect on thicknesses, phase transition temperature, or morphology of the bilayer was observed during fullerene movement [46]. Other studies found that fullerenes with polar functional groups, such as C_60_(OH)_20_ and fullerene derivatives like tris-malonyl-fullerene, did not enter the membrane but rather remained at the lipid bilayer/water interface [27,31,47]. In contrast, the penetration of Janus fullerene (half polar and half hydrophobic beads) into regular and fully peroxidized bilayer was observed [32,37]. Gul et al. [37] found that Janus fullerenes translocate towards the head groups of both regular and fully peroxidized bilayers. Sridhar et al. reported that fullerenes quickly crossed the regular membrane and settle in the opposite head groups [32]. Therefore, the ability of fullerenes to penetrate biological membranes can depend on various factors, including the presence of polar functional groups, the size of fullerene, the ability of fullerene to cluster, and the lipid types making up the membrane (single-lipid bilayers, binary mixtures, - multicomponent lipids and peroxidized membranes). Understanding the free energy profile of a fullerene translocating from water into the bilayer can help to rationalize the process.

The potential of mean force (PMF) for fullerene penetration into the bilayer can be calculated using the umbrella sampling method [48,49]. The free energy of transferring a fullerene decreases as it moves from the water phase into the membrane and reaches the lowest value in the membrane region. Notably, there is no free energy barrier for the transport of fullerenes from the water phase into the lipid core of the bilayer [38]. The effectiveness of fullerene penetration into lipid bilayers is determined by the hydrophobicity and size of the fullerene [24,30]. However, the other factors such as the specific chemical properties and structure of the fullerene may also influence the ability of fullerenes to penetrate lipid bilayers [50].

## The location of fullerene in biological membrane

**Figure 2.**
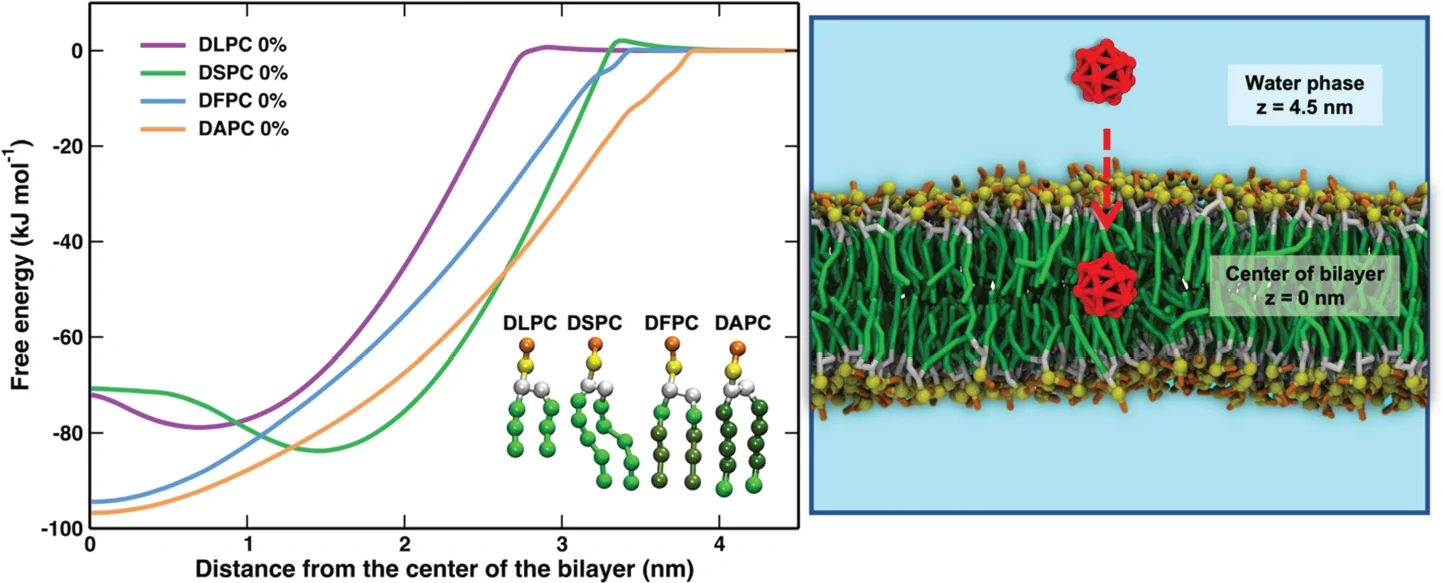
Potential of mean force for a fullerene translocation across saturated (DLPC, DSPC) and unsaturated (DFPC, DAPC) lipid bilayers from the water phase into the center of the lipid bilayers as a function of distance in the z-direction. Adapted from nisoh et al [20].

Fullerenes were found to be stably located inside lipid bilayers, raising the question of where they are positioned within the membrane. These issues must be thoroughly investigated at the molecular level to comprehend the interaction of fullerene with lipid tails. MD simulations have shown that the behavior of fullerenes inside lipid bilayers depends on the concentrations of fullerenes and types of lipids [20,36,39,40,51,52]. At low concentration of fullerenes in the single saturated bilayers made of DLPC, DPPC or DSPC, fullerenes prefer to be located in the lipid tail region (Figure 1(a)). Figure 2 shows that the locations of free energy minimum for DLPC and DSPC lipid bilayers are at 0.7 and 1.4 nm from the center of bilayer, respectively. These findings are supported by all-atom [27,28] and other CG-MD simulations of DPPC and DMPC lipid bilayers [30,53]. At high concentration, the density profile of fullerenes in the bilayer can be separated in three regions, including the center of the bilayer and two hydrocarbon tails (Figure 1(a)) [20,51]. This suggests that fullerene interacts with lipid tails rather than lipid headgroups. In the case of unsaturated lipid bilayers (DOPC, DVPC, DUPC and DFPC lipids), fullerenes were found to be stably located in the center of the bilayer to avoid contact with double bond region of lipid tail. The avoidance of the double bond region of lipids by fullerene can be directly observed from the snapshots and density profiles of fullerene in the bilayers (Figure 1(a)). Fullerene is preferentially located in the lipid tail region and bilayer’s center for saturated and unsaturated lipid bilayers, respectively. These findings are consistent with our potential of mean force (PMF) calculations, as depicted in Figure 2. Additionally, the difference in the relative position of fullerene in lipid bilayers upon the location of double bond is also supported by PMF of DFPC and DAPC (the highly unsaturated lipids). The positions of unsaturated beads are near the end of the tail and the head for DFPC and DAPC, respectively. In the DFPC bilayer, the PMF shows a distinctive wrinkle at ~3.2 nm from the bilayer center, indicating fullerene localization near the head of lipids. In contrast to the PMF of DAPC, the energy profile monotonical decreases towards the bilayer center. These observations indicate that fullerenes interact less favorably with double bonds, which drives fullerenes towards the central region of the membrane. Interestingly, the energy stabilization of the highly unsaturated DFPC bilayer was found to be the highest among all lipid types, at 94.4 kJ/mol. This finding is consistent with Sastre et al. in DUPC lipid bilayer [36]. As the fullerene concentrations increased in both saturated and unsaturated bilayers, they become less stable inside the bilayer. This can be explained by a reduction in free volume inside the bilayer as the density of fullerene increase. In addition, the location of fullerenes in the plasma membrane has also been investigated [19]. In the latter, fullerene prefers the inner leaflet of the plasma membrane in the regions with highly unsaturated lipids and avoid the regions containing cholesterols and mono- and di-unsaturated lipids. These findings suggest that fullerenes can be loaded deeply within the plasma membrane, which could be useful for the design of drug carrying liposomes. According to the simulation results, the saturation level of the tail lipid group play an important role on the localization of fullerenes within the membrane.

## Aggregation and dispersion of fullerenes inside biological membrane

**Figure 3.**
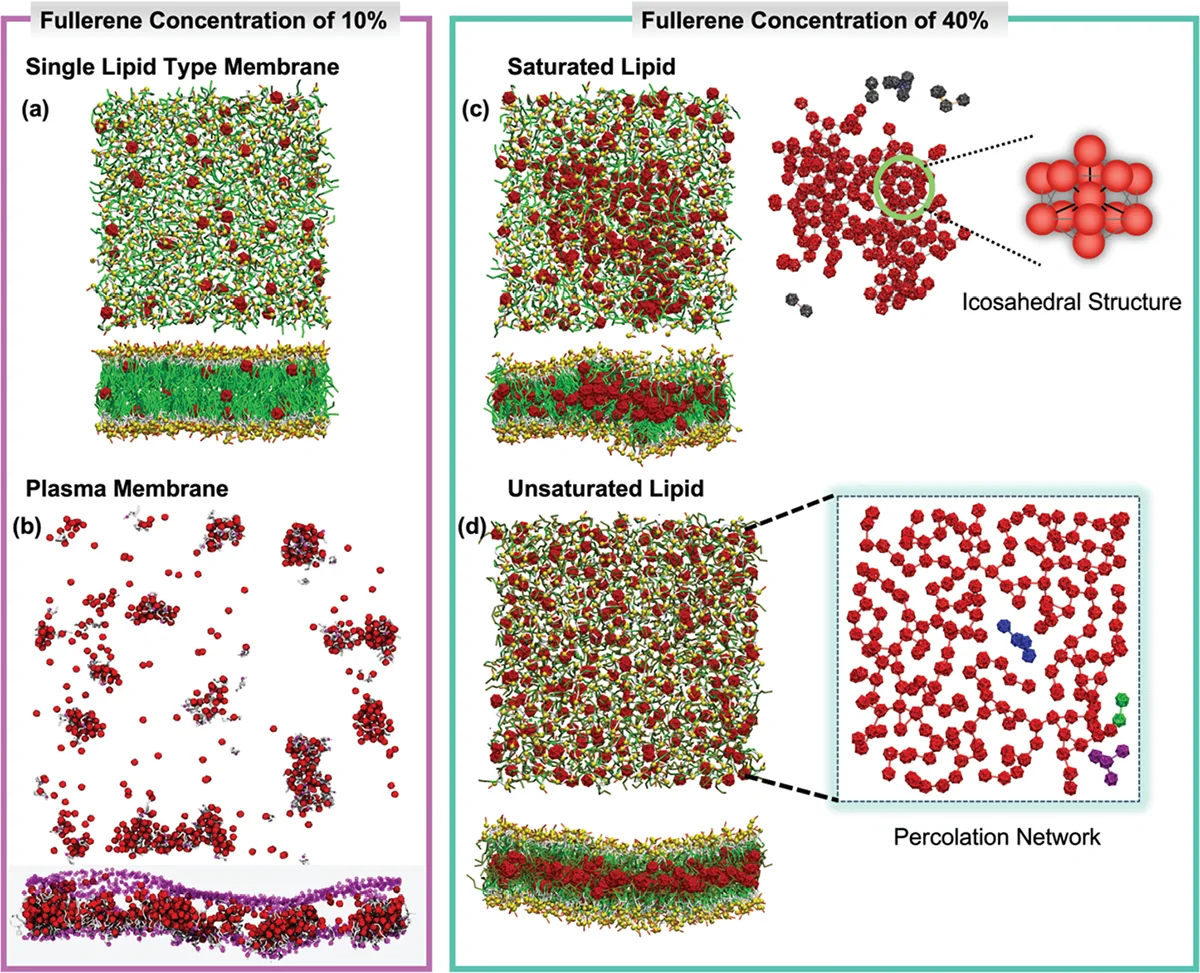
The behavior of fullerene in membranes with varying fullerene concentrations and lipid saturation levels. (a) Fullerene dispersion in a single lipid type bilayer at a concentration of 10%, taken from Nisoh et al [20]. (b) Fullerene aggregation in the plasma membrane at a concentration of 10%, reproduced with permission from Nisoh et al [19]. (c) Fullerene aggregation in a saturated lipid bilayer at a concentration of 40%, adapted from nisoh et al [20]. (d) Fullerene aggregation in an unsaturated lipid bilayer at a concentration of 40%, adapted from Nisoh et al [20].

In addition to the location of fullerenes in the membrane, questions have been raised regarding to the ability of fullerene to aggregate or disperse within lipid bilayers and the physical mechanisms controlling the behavior of fullerene aggregation. It is known that fullerenes aggregate in water forming clusters with an average diameter ranging from 10 to 100 nm [54–58]. In contrast, fullerene remains disperse in hydrophobic solvents [59,60]. Nonetheless, experimental results on fullerene aggregation in membranes remain contradictory, especially considering the unknown sizes of aggregates [61,62]. Back in 2008, Wong-Ekkabut et al. demonstrated that after penetration, fullerenes did not aggregate in the DOPC bilayer interior [30] (Figure 3(a)). Other simulations, using an atomistic model of fullerenes and DMPC bilayer revealed dispersion phenomena in the bilayer interior [28,38,63]. The dispersion behavior can be explained using fullerene thermodynamics and kinetics. Free energy analyses indicate that a dimer is energetically unfavorable due to distortions in the bilayer structure [28,38,64]. In addition, a study by Li et al. showed that the interaction between two fullerene molecules in the bilayer is less attractive than in an alkane with the same molecular weight as the lipid chains [28]. Several experiments are in agreement with the MD simulations, indicating that fullerene aggregation cannot be observed in lipid membranes [65–68]. The knowledge of these findings has been applied to the development of liposomes designed to dissolve and deliver fullerenes to cells [65,69]. In these systems, fullerene molecules are incorporated into the liposome bilayer, and the chemical affinity can be modified by varying the lipid compositions.

On the other hand, there is evidence of fullerene aggregating strongly in lipid bilayers. Fullerenes tend to aggregate after being steered into the membrane even at very low concentration [28,29,70]. At high concentration, the aggregation of fullerene molecules in lipid membranes are also supported by several experimental and computational investigations [24,36,40,51,52,61,71–74]. However, fullerene aggregation in bilayers at high concentration remains a crucial open question. In 2019, we proposed a thermodynamic explanation for how fullerenes favors dispersion and aggregation in lipid bilayers at low and high fullerene concentrations, respectively [51]. These different behaviors emerge from the balance between entropy and enthalpy of fullerene aggregation. Fullerenes in fully saturated DPPC bilayers are controlled by the enthalpic part, which favors aggregation at concentrations above 20%. The cases of DOPC (single double bond in both of the acyl chains) and POPC (as in Barnoud et al.) [64] bilayers show that entropy dominates and fullerenes favor dispersion even at high concentrations. In addition to improving the fundamental understanding of the aggregation state, Nisoh et al. performed a series of systematic analyses using seven different bilayers (with varying lipid saturations and tail lengths) and five fullerene concentrations [20]. This systematic study suggested that the aggregation behavior of fullerene in lipid bilayers is not only dependent on the fullerene concentration but also related to the lipid composition of the bilayer in the term of the saturation levels and acyl chain lengths.

Furthermore, fullerenes in real biological membranes may aggregate even at low fullerene concentrations (Figure 3(b)) [19]. This finding is supported by the discovery of fullerene aggregations in the plasma membrane using electron microscopy [17]. Investigations of fullerene aggregation can rationalize their potentially harmful effect on biological membranes and provide insights into the characteristics of fullerene aggregation within membranes. The toxicity of fullerene aggregation is discussed later in the section on the effects of fullerenes on membranes.

## The geometry of fullerene clusters in water and bilayer

The structural and dynamic properties of fullerenes in water have been studied using both all-atom [75] and coarse-grained models [39]. Free energy calculations were performed to investigate the aggregation of fullerenes in water, revealing that the interaction between fullerenes is dominated by direct fullerene–fullerene interactions rather than water-induced hydrophobic interactions in all cluster geometries. Furthermore, the geometry of the fullerene cluster in the membrane was investigated [20], discovering that the formation of fullerene aggregates in membranes is influenced by lipid types. In saturated (DLPC, DPPC, DSPC) and monounsaturated (DOPC, DVPC) lipid bilayers, fullerenes aggregate and form clusters of icosahedral structures (Figure 3(c)). However, in polyunsaturated bilayers (DUPC and DFPC), the fullerene molecules form a network at the middle of the bilayer (Figure 3(d)).

Interestingly, when fullerene molecules aggregate in lipid bilayer, a cluster forms Mackay’s icosahedral structure, which is a stack of layers with five-fold symmetry [76]. This structure was first identified using mass spectrometry [77] and lately confirmed by computational approaches [78–82]. Additionally, the geometry of fullerene clusters in aqueous solution is similar to that within a lipid bilayer [75]. Nisoh et al. has also found that the five-fold symmetry plane of fullerene clusters in a lipid bilayer is aligned with the bilayer plane, whereas in aqueous solution, it is random. These results demonstrate the symmetry plane of fullerene clusters is not affected by the pressure coupling method, whether it is isotropic or semi-isotropic [20]. It should be noted that the situation is a little more complicated at high concentration of fullerene in a lipid bilayer because of the confined environment.

## Effect of fullerenes on biological membranes

Although experimental studies [71,83–85] suggest that pristine fullerene has no significant effect on membrane stability, toxicity or lethality in cells, several simulation studies report that the entry of pristine fullerene molecules into the biological membrane could affect its physical and mechanical properties [20,30,36,40,51]. Changes in the properties of the bilayer are clearly affected by the dispersion and aggregation of fullerenes, which in turn depends on the concentrations of fullerenes and the types of lipids. Understanding the mechanism of lipid bilayer perturbation by fullerenes is critical in determining their potential harmful effect on biological membranes.

At low concentration, fullerene accounts for only a minor effect on the physical properties of the lipid bilayer including area per lipid, volume per lipid, bilayer thickness and order parameter [30,35,40,51,70]. A study using NMR experiments also found that fullerene did not significantly change the order parameter of lipids at low concentration [24]. Moreover, physical membrane disruption or rupture was not observed in the peroxidized bilayer [37]. However, perturbation of the physical structure of the bilayer became significantly pronounced at high fullerene concentration. Fullerene was found to change the membrane structure and induce undulations [40,67]. Aggregated fullerenes interact strongly with lipid bilayers and cause a decrease in their mechanical stability leading to membrane bending and deformation [20,36,40,51]. Moreover, fullerene clusters induce bilayer curvature and the occupation of hydrophobic regions, potentially increasing the membrane’s vulnerability to attack by reactive oxygen species (ROS) [31].

In general, structural properties such as area per lipid, volume per lipid, and thickness increase monotonously with increasing fullerene concentration. This trend is consistent with x-ray measurements using multilamellar POPC vesicles with varying fullerene concentrations [67]. However, the thickness of saturated lipid bilayers such as DLPC, DPPC and DSPC exhibits non-monotonous behavior, decreasing with increasing fullerene concentration and then increasing at high concentrations [20,51]. One possible explanation is the behavior of fullerene, which begin to aggregate at high concentrations [20,51]. To understand how fullerene aggregation affects bilayer properties, the detailed properties of a lipid molecule were analyzed. For example, the lipid tilt angle is the angle between lipid vector and z-axis. In saturated bilayer, fullerene located in the acyl chain region causes a large tilt angle and a decrease in bilayer thickness. At higher concentrations, fullerene aggregation changes the lipid tilt and leads to an increase in bilayer thickness. These changes are related to the thickness of saturated bilayer. In the case of unsaturated bilayer, fullerenes are located at the center of bilayer, causing the lipid chains to stretch and creating free space for fullerenes. In addition, the tilt modulus of lipid bilayer in the absence of fullerene has been studied through atomistic simulations [86,87]. The tilt modulus was found to increase with increasing concentration and to have a significant effect on fully saturated bilayers [19]. Moreover, the local 2D density and thickness have shown that the increase in bilayer thickness is directly correlated with the location of fullerene aggregations [19,20]. Therefore, fullerene aggregation has a strong influence on lipid bilayers with different degrees of acyl chain saturation and chain length. When fullerenes aggregate, their interactions with the lipid tails change.

**Figure 4.**
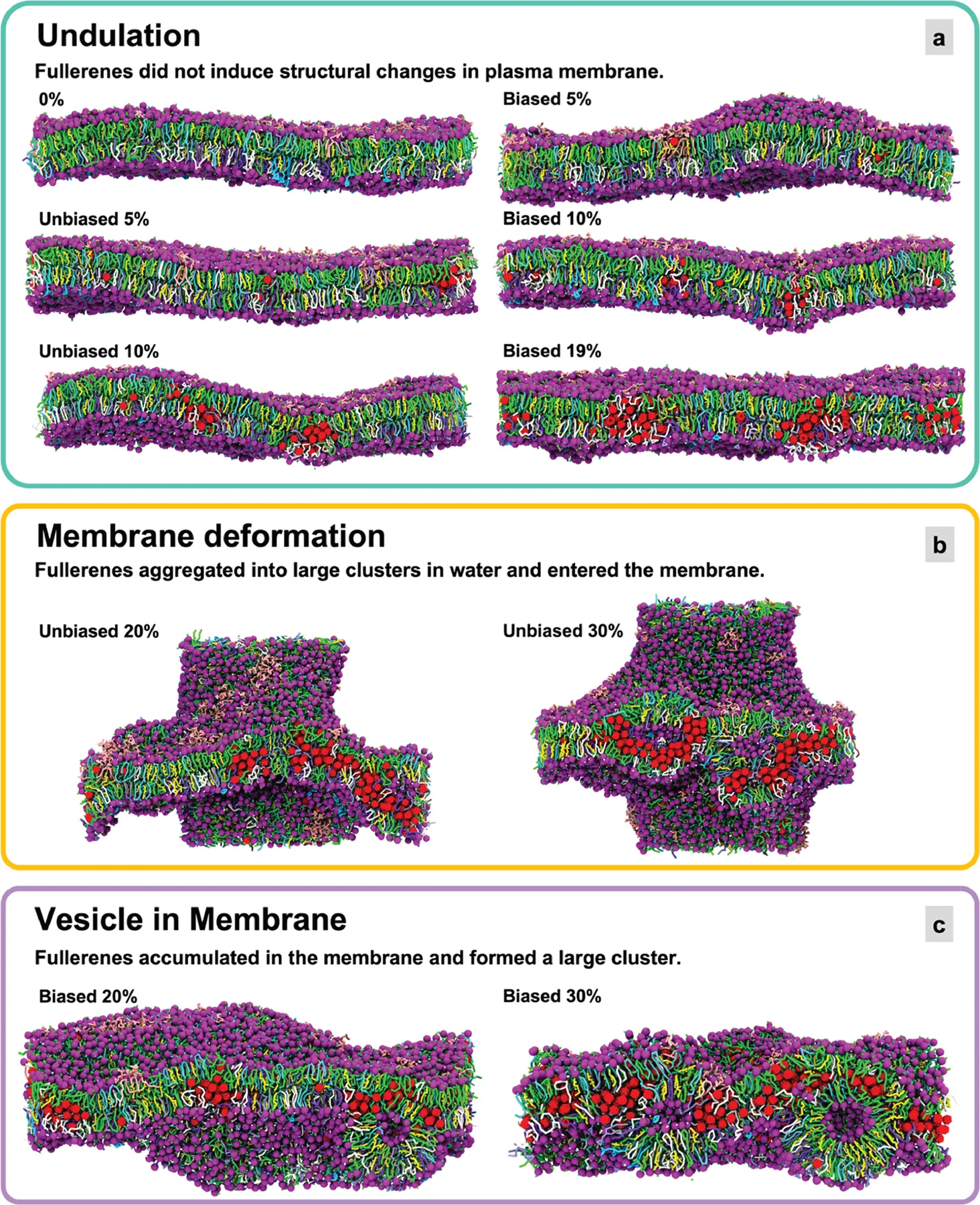
Side view of the plasma membrane with incorporated fullerenes, reproduced with permission from Nisoh et al [19]. (a) Undulation in the plasma membrane. (b) Plasma membrane deformation. (c) Vesicle in the plasma membrane.

The effect of fullerene aggregation on complex membranes was also studied [19]. Deformation of the plasma membrane are shown in Figure 4. Despite fullerene aggregation taking place only at high concentration in the inner leaflet region, undulation of the plasma membrane was observed even at low fullerene concentrations (Figure 4(a)). Membrane damage was observed with increasing fullerene concentration. The formation of large fullerene aggregates in water caused membrane deformation during its insertion in the bilayer (Figure 4(b)). Moreover, the accumulate fullerenes on the bilayer were observed to grow into large clusters that nucleated into small vesicles (Figure 4(c)). It should be noted that the presence of vesicle might disturb the transport of small molecules, resulting in loss of membrane function and disrupt membrane function.

Considering membrane damage, some studies have suggested that fullerene does not aggregate within lipid bilayers and does not cause physical damage [30,51]. However, other studies have found that the accumulation of fullerene on cell membranes leads to their aggregation at high concentrations. The aggregated fullerenes cause changes on the mechanical properties of the bilayer [19,39,40,52,61]. An increase in fullerene cluster size in the water phase leads to bilayer rupture because the size of clustered fullerenes exceeds bilayer thickness [39,68]. In addition, the presence of large fullerenes such as C_180_ and C_540_ causes strong curvature on bilayers [29,88]. Experimental studies have demonstrated that oxidative stress induced by fullerene aggregation could be extremely toxic for fish, human skin, liver cells, and lung tissue [24,61,71,89]. Additionally, vesicles rupture, membrane leakage and cell death were caused by the aggregation of fullerene. Considering both experimental and simulation factors, the determination of fullerene toxicity on living cell remain challenging. A number of studies illustrate the potentially harmful effect of fullerenes on biological systems and also determine the mechanisms of how fullerene disturb membrane. It is anticipated that a better understanding of the interaction between fullerenes and membranes will lead to novel designs of fullerene systems for nanomedicine applications including drug delivery systems and anticancer agents [19,35,61]. In the same vein, encapsulating fullerene in bilayer vesicles like liposome may be used for applications in therapeutics and molecular imaging [90,91]. Fullerene concentration and lipid composition can be varied to reduce side effects and improve treatment efficacy [24,92,93].

## Perspective and future works

Given that the interaction of fullerenes with biological membranes can account for toxicity at the cellular level, it is critical to provide an atomic-level understanding of these interactions. This has been provided in recent years by experimentally guided computer simulations. Fullerenes are promising molecules for various applications including drug delivery systems wherein their properties are strongly affected by lipid composition and fullerene concentration.

Most studies have already focused on fullerenes in single component bilayer or binary mixtures, as well as peroxidation lipid membranes. Adding to previous studies, our group reported on the behavior of fullerene in both saturated and unsaturated lipid bilayers and how this behavior is affected by fullerene concentration. These studies suggest that the lipid saturation level is a key factor in determining how fullerenes aggregate and where they localize within the lipid bilayer. This information is critical to rationalize fullerene’s behaviour in different single type of lipid bilayers. This type of understanding is also crucial for the development of rational models to predict real biological membranes. A level up in the reality of computational studies can be achieved by increasing the diversity of lipid types in cell membranes. Fortunately, a plasma membrane model was successfully developed by Ingolfsson et al [43], which we used to provide the first insights into fullerene’s effect on the plasma membrane. We anticipate that results from this study will be followed up by additional experimental and computational investigations to unravel the toxicity of fullerenes on real biological membranes. This expectation was recently confirmed by an experimental study using electron microscopy [17]. The translocating behavior of fullerene through plasma membranes remains still unclear and it should be investigated further in the near future. This may include calculating the energy profile of fullerene as it interacts with plasma membranes. This information is critical to understand fullerene’s localization in the plasma membrane, which corresponds to the minima in the free-energy profile. Future endeavors in our lab are along those lines.

## Abbreviations

C_60_: Buckminsterfullerene
MD: Molecular Dynamics
CG: Coarse – grained
PMF: Potential of Mean Force
NMR: Nuclear Magnetic Resonance
ROS: Reactive Oxygen Species
DPPC: Dipalmitoyl – Phosphatidylcholine
DMPC: Dimyristoyl – Phosphatidylcholine
DOPC: Dioleoyl – Phosphatidylcholine
DLPC: Dilauroyl – Phosphatidylcholine
DSPC: Distearoyl – Phosphatidylcholine
DFPC: Dioctadecatrienoyl – Phosphatidylcholine
DAPC: Diarachidoyl – Phosphatidylcholine
DVPC: Divaleryl – Phosphatidylcholine
DUPC: Dilinoleoyl – Phosphatidylcholine
POPC: Palmitoyloleoyl – Phosphatidylcholine
